# Information Safety Assurances Increase Intentions to Use COVID-19 Contact Tracing Applications, Regardless of Autonomy-Supportive or Controlling Message Framing

**DOI:** 10.3389/fpsyg.2020.591638

**Published:** 2021-01-07

**Authors:** Emma L. Bradshaw, Richard M. Ryan, Michael Noetel, Alexander K. Saeri, Peter Slattery, Emily Grundy, Rafael Calvo

**Affiliations:** ^1^Institute for Positive Psychology and Education, Australian Catholic University, North Sydney, NSW, Australia; ^2^School of Health and Behavioural Sciences, Australian Catholic University, Brisbane, QLD, Australia; ^3^Monash Sustainable Development Institute, Monash University, Melbourne, VIC, Australia; ^4^Dyson School of Design Engineering, Faculty of Engineering, Imperial College London, London, United Kingdom

**Keywords:** coronavirus, autonomy, information security, self-determination theory, controlling, message framing

## Abstract

Promoting the use of contact tracing technology will be an important step in global recovery from the COVID-19 pandemic. Across two studies, we assessed two messaging strategies as motivators of intended contact tracing uptake. In one sample of 1117 Australian adults and one sample of 888 American adults, we examined autonomy-supportive and controlling *message framing* and the presence or absence of *information safety* as predictors of intended contact tracing application uptake, using an online randomized 2 × 2 experimental design. The results suggested that the provision of data safety assurances may be key in affecting people’s intentions to use contact tracing technology, an effect we found in both samples regardless of whether messages were framed as autonomy-supportive or controlling. Those in high information safety conditions consistently reported higher intended uptake and more positive perceptions of the application than those in low information safety conditions. In Study 2, we also found that perceptions of government legitimacy related positively to intended application uptake, as did political affiliation. In sum, individuals appeared more willing to assent to authority regarding contact tracing insofar as their data safety can be assured. Yet, public messaging strategies alone may be insufficient to initiate intentions to change behavior, even in these unprecedented circumstances.

## Introduction

Countries around the world continue to experiment with policy responses to manage COVID-19 infections and harms, often to greater and lesser effectiveness and tolerability among their citizens. Countries and regions seeking to reduce strict social distancing measures (i.e., stay at home orders) must find alternative methods of managing the spread of infection. One effective way to do so may be to trace the contacts of people who are COVID-19 positive, and test those contacts. The process typically involves laboriously interviewing the infected person to identify possible contacts. Contact tracing technologies can rapidly accelerate this process. With contact tracing, people can use software on their mobile devices to track their recent contacts. Health professionals can then use the software to notify those who have been in close contact with a newly infected person, so those at risk can get tested or self-isolate. However, the effectiveness of the application will be commensurate with its community uptake. If very few people use the technology, its effectiveness will be greatly compromised. Therefore, understanding how to best motivate use of contact tracing applications is of vital importance to the process of recovering from the COVID-19 pandemic. *Self-determination theory* (SDT; Ryan and Deci, 2017) provides a parsimonious and evidence-based framework for understanding how the framing and content of social messages can motivate or undermine behavior change.

Evidence from SDT finds that environments that support feelings of meaning, volition, and choice—that is, environments that support autonomy—facilitate the internalization of ambient values (Ryan and Deci, 2017; Nishimura et al., 2020), and can promote positive, healthy decision making (Williams et al., 2006). In contrast, when people feel subject to external controls or inductions that are controlling, individuals often show less willingness to adopt or maintain the target behaviors (Vansteenkiste et al., 2005; DeCaro and Stokes, 2008) and may even reject imposed values (Hawley et al., 2002). Indeed, the provision of autonomy-support has been meta-analytically linked to greater sense of value for and adherence to a host of health-related behaviors over time (Gillison et al., 2019; Ntoumanis et al., 2020). Across two studies, we experimentally manipulate two elements of social messaging expected to impact people’s willingness to download a COVID-19 contact tracing application. The first strategy uses an autonomy-supportive versus controlling message framing to promote use of the application; the second uses messaging inferring high or low levels of information privacy, non-surveillance, and safety.

It is well established that prolonged exposure to autonomy-support and control influences behavior (Ng et al., 2012; Slemp et al., 2018). However, the effect of autonomy-supportive and controlling social messages on promoting new behaviors has been less researched. Some prior research suggests that autonomy-supportive messages may be more persuasive than messages framed with controlling language (Legault et al., 2011). Autonomy-supportive messages provide a meaningful rationale for a recommendation and minimize feelings of pressure by emphasizing individual choice (e.g., Jang, 2008) thereby promoting behavior endorsement due to identified value, rather than external pressure (Ryan and Deci, 2017; Vansteenkiste et al., 2018). In contrast, messages with a controlling framing attempt to induce guilt or pressure by using words like “should” and “must,” which can prompt behavior, and yet diminish individuals’ feelings of autonomy, often resulting in resistance to or even defiance of the message (Legault et al., 2011). In sum, autonomy-supportive message framing may allow individuals to better identify with messaging goals, thereby increasing the likelihood of adherence to recommendations relative to controlling messages. In the context of COVID-19 tracing applications, uptake should thus be more encouraged by autonomy-supportive than by controllingly-framed messages.

Although the potential utility of contact tracing is self-evident, the use of such technologies also raises other issues regarding psychological experiences of autonomy, most notably the potential for surveillance and fears of loss of control of personal information (Calvo et al., 2020). Indeed, past studies show that experiences of surveillance can undermine a sense of autonomy and decrease motivation for behavior (e.g., Plant and Ryan, 1985; Enzle and Anderson, 1993). Concerns about the storage and use of data collected by COVID-19 contact tracing applications may thus lead to lower adoption if potential users cannot be assured that their activities will not be surveilled for other purposes and that their data are fully protected. We thus expected that making data safety assurances salient would result in greater intention to uptake the application, relative to a condition where data protection is less transparent or guaranteed. While such a claim may seem intuitive, when the content of far-reaching and influential public health messaging is at stake, evidence for intuitions is essential.

Data safety assurances are important in promoting public health compliance because such declarations map on to people’s inherent need to feel psychologically safe and free from government surveillance and control (Calvo et al., 2020). In addition, data safety relates to perceptions of authority as being legitimate and trustworthy, and perceived legitimacy of authority is related to more autonomous compliance (Ryan and Deci, 2017). For example, Graça et al. (2013) showed that adolescents’ deference to teacher authority and willingness to follow rules was higher when the teacher was perceived as generally autonomy-supportive. Therefore, we also expected, and test in Study 2, that perceived government legitimacy would also be associated with greater willingness to uptake contact tracing. Testing the aforementioned hypotheses was the central goal of the ensuing studies.

## Study 1

Using a large samples of adults from Australia, in Study 1, we examined three primary effects: (1) The impact of autonomy-supportive and controlling message framing in promoting positive perceptions of, and intentions to use, a contact tracing application; (2) The impact of information safety messages in promoting positive perceptions of, and intentions to use, a contact tracing application; and (3) The interaction between message framing and information safety in promoting positive perceptions of, and intentions to use, a contact tracing application. Using a 2 × 2 factorial analysis of covariance (ANCOVA), we expected to find a main effect of *message framing*, such that participants in the autonomy-supportive conditions would report more positive perceptions of the application than those in the controlling groups. Similarly, we expected to find a main effect of *information safety*. Specifically, we expected participants in the high information safety condition to be more in favor of the application than those in the low information safety condition. The hypotheses for this study were preregistered on the Open Science Framework https://osf.io/q7mju/.

### Method

#### Participants

The sample comprised 1117 Australian adults, recruited by a professional panel company. Participants completed the survey online. The age range of the sample was 18–89 (*M* = 50.17, *SD* = 17.46). We did not collect additional demographic information in this survey. Applying sensitivity analyses (Perugini et al., 2018) in G^∗^Power (Faul et al., 2007), we evaluated the minimum detectable effect size given our analytic strategy, 1117 sample size, an alpha level of 0.05, power of 0.08, and one covariate (prior intention to download the application). The results suggested that our *N* of 1117 was sufficient to reliably detect effects as small as η_*p*_^2^ = 0.01 (reflected in a critical *F* statistic of 3.85). G^∗^Power derives an *f* statistic effect size in sensitivity analysis (in this case the *f* value was 0.083), so we used the formula reported in Cohen (1988, p. 281) to convert *f* to η_*p*_^2^ (simply *f*^2^), and rounded to the second decimal place.

#### Materials

Our study materials were presented with a battery of other items for the purposes of separate studies. We did not refer to nor preregister hypotheses related to the other variables in the study and so do not mention them here. More details about the complete questionnaire battery can be found here [link available here: https://osf.io/u5x3r/].

##### Pre-experiment items

###### Likelihood of using the application

We expected that participants’ initial likelihood of downloading a contact tracing application would be a substantial predictor of their post-experiment intentions to download. Therefore, to control for initial intentions we posed the question “How likely are you to download and install a government COVID-19 tracing app on my phone?” The item was responded to on a 0 (not at all likely) to 10 (extremely likely) scale.

##### Post-experiment items

###### Perceptions of contact tracing applications

We posed three post-experiment questions to assess participants’ perceptions of a COVID-19 contact tracing application: (1) How likely would you be to download and install a COVID-19 tracing app? (0 = not at all likely–10 = extremely likely); (2) Do you think a COVID-19 tracing app is a good idea for your government to fund? (0 = extremely bad idea–10 = extremely good idea); and (3) How likely is it that you would recommend a COVID-19 tracing app to a friend, family member, or colleague? (0 = not at all likely to recommend–10 = extremely likely to recommend). We also presented participants with five additional questions related to their valuing of the application, trust for the application, perceived usability of the technology, and their self- or other-focused reasons for using the application. However, we did not pre-register hypotheses pertaining to these items, so we present these items in Online Supplementary Material S2 and their correlations with the rest of the study variables in Online Supplementary Material S3.

##### Experimental manipulation

After answering the pre-experiment questions, participants were randomly assigned to one of four conditions: autonomy-support with high information safety (*n* = 268), autonomy-support with low information safety (*n* = 262), control with high information safety (*n* = 303), and control with low information safety (*n* = 284). Participants were naïve to their condition as were experimenters because the study was conducted online. All participants were presented with the same introduction, followed by a condition-specific combination of two of four possible vignettes, we present the condition-specific vignettes below (with the full experiment available in Online Supplementary Material S1). The autonomy-support and control vignettes were word count-matched at 128 words each, as were the information is safe and information is not safe conditions at 84 words each.

###### Autonomy support condition

Downloading the COVID-19 trace app means you are allowing information about who you have come into close contact with to be electronically monitored, which may feel intrusive. The reason for this unusual measure is that it is the most effective way to help people find out about their risk if they have come in contact with an infected person. Doing so means they can then make the right choices to protect themselves and their loved ones. That is why it is hoped that you will choose to participate in this important program. Using the app is entirely voluntary. You have the choice to download and to activate, and you can opt out at any time. Making this choice is a way you can really contribute to containing the spread.

###### Controlling condition

Downloading the COVID-19 trace app means you are allowing information about who you have come into close contact with to be electronically monitored. Even if it feels intrusive, this is something people should not question, because it is clearly the most effective way for authorities to track who has been in contact with an infected person. You need to help authorities notify those at risk of contracting the virus. Given the current threat, we think you must do this to be a responsible citizen. Downloading the app is not really a choice—it is a thing that you should just do. To comply with this program, you should download the app and ensure that it is activated. Complying with this requirement is the best way to stop the spread of the virus.

###### High information safety condition

Information from the COVID-19 trace app will be stored locally on a phone, encrypted, and only transferred to a health data bank if a person tests positive for COVID-19. Once there, data cannot be accessed by any other parties, private or governmental, and will not be used for any other purposes. The app is designed so that your personal identity and personal information are protected. Data will be destroyed every 21 days so that it cannot be used later by anyone, for any reason.

###### Low information safety condition

Information from the COVID-19 trace app will be stored locally on a phone and then transferred to a health data bank for use in tracing the contacts of a person who tests positive for COVID-19. Once there, the data will be owned by the government and may be accessed for other important purposes. The app is designed so that the data can be stored long-term and it is possible that the data will be used in later analyses for other health or government purposes.

### Results

#### Preliminary Analysis

All analyses (in Studies 1 and 2) were conducted in R Version 3.6.0 (R Core Team, 2019), using packages including dplyr (Wickham et al., 2019), corx (Conigrave, 2019), psych (Revelle, 2017), sjstats (Lüdecke, 2020), and lsr (Navarro, 2015). Means, standard deviations, and Pearson’s correlations between the study variables are included below in Table 1. As we expected, pre-experiment intention to download a COVID-19 contact tracing application correlated strongly with post-experiment intentions, and positive post-experiment perceptions of a contact tracing application were sensibly positively associated. Correlations reported in Online Supplementary Materials S2, S3 demonstrate that seeing value in the application, trusting its safety, and seeing it as beneficial to oneself and to others, were all strongly positively correlated with intention to download and use the application across conditions.

**TABLE 1:** Inter-correlations, means, and standard deviations for the variables in Study 1.

	1	2	3	4
1. Pre-test likelihood of downloading	–			
2. Post-test intention to download	0.79***	–		
3. Post-test government should fund	0.61***	0.77***	–	
4. Post-test recommend to others	0.68***	0.85***	0.81***	–
Mean	3.96	4.60	5.85	4.94
SD	3.49	3.57	3.05	3.38

Note. ***p < 0.001. Pre-test, pre-experiment; Post-test, post-experiment.

We were not able to reliably detect any meaningful differences across the four groups in either pre-experiment likelihood of downloading the application, *F*(3,1111) = 1.09, *p* = 0.35, η_*p*_^2^ = 0.003, or in mean age, *F*(3,1113) = 0.13, *p* = 0.95, η_*p*_^2^ = 0.00. The small number of participants who did not respond to all items (range from 0.18 to 1.97% missing responses across the pre- and post-experiment variables) was omitted from the relevant analyses. All dependent variables were standardized prior to analysis.

#### Primary Analysis

To test if the total participant reports of likelihood of downloading the COVID-19 contact tracing application increased from pre- to post-experiment, we conducted a paired samples *t*-test, which indicated that there was a statistically significant increase in likelihood/intention to download the COVID-19 contact tracing application from pre- (*M* = 3.96, *SD* = 3.49) to post-experiment (*M* = 4.60, *SD* = 3.57), *t*(1113) = −9.35, *p* < 0.001 [95% CI −0.78, −0.51], Cohen’s *d* = 0.28. We also examined change from pre- to post-intention to download within each experimental group. There were small to moderately-sized, statistically significant increases in intention to download from pre- to post-experiment in all four experimental groups: (1) the autonomy-support plus high information safety group, *t*(265) = −5.79, *p* < 0.001 [95% CI −1.18, −0.58], Cohen’s *d* = 0.35, (2) the autonomy-support plus low information safety group, *t*(261) = −3.57, *p* < 0.001 [95% CI −0.80, −0.23], Cohen’s *d* = 0.22, (3) the control plus high information safety group, *t*(302) = −6.73, *p* < 0.001 [95% CI -1.10, −0.60], Cohen’s *d* = 0.37, and (4) the control plus low information safety group *t*(282) = −2.49, *p* < 0.01 [95% CI −0.57, −0.07], Cohen’s *d* = 0.15 (see Table 2 for group-specific means and standard deviations).

**TABLE 2:** Experimental group-specific means and standard deviations for the pre-experiment (pre-test) and post-experiment (post-test) measures in Study 1.

	Aut + Safe	Aut + Not Safe	Cont + Safe	Cont + Not Safe
Pre-test likelihood	3.84 [3.40]	3.72 [3.41]	4.01 [3.56]	4.23 [3.56]
Post-test intentions	4.71 [3.42]	4.24 [3.48]	4.86 [3.65]	4.55 [3.68]
Post-test support	5.97 [2.92]	5.68 [2.98]	5.90 [3.05]	5.84 [3.25]
Post-test recommend	5.04 [3.18]	4.46 [3.46]	5.26 [3.41]	4.95 [3.43]
Pre-post difference	0.88 [2.49]	0.52 [2.34]	0.85 [2.20]	0.32 [2.15]

*Note. Aut, autonomy-supportive message framing condition; Cont, controlling message framing condition; Safe, high information safety condition; Not Safe, low information safety condition; Pre-test likelihood [to download the application]; Post-test intentions [to download the application]; Post-test support [the government investing in the application]; Post-test recommend [the application to friends, family, and colleagues]; Pre-post difference, post-test intentions minus pre-test likelihood.*

Next, to examine the roles of message framing and information safety assurances in predicting group differences on the post-experiment measures, we ran three 2 × 2 factorial ANCOVAs using the two (message framing and information safety) two-level (autonomy versus control and high information safety versus low information safety) factorial predictors. First, we predicted post-experiment intention to download the application. Second, we predicted post-experiment perceptions of the application as a worthwhile use of government resources. Third, we predicted post-experiment intention to recommend the application to friends and family. In all three models, we controlled for self-rated initial likelihood of downloading the application.

##### Autonomy-supportive versus controlling message framing

**FIGURE 1:**
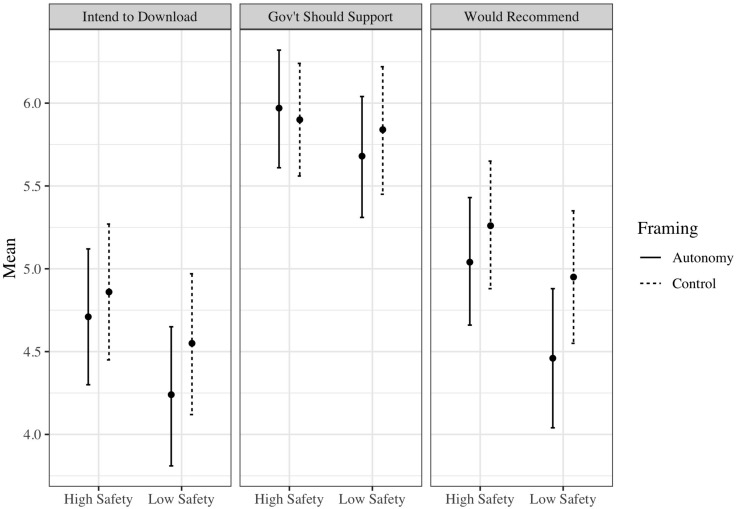
Mean scores and 95% confidence intervals for the three dependent variables according to message framing condition (autonomy or control) by information safety condition (high or low) in Study 1.

The experimental group-specific means presented in Table 2 (and illustrated in Figure 1), coupled with the 2 × 2 factorial ANCOVA results shown in Table 3, demonstrate that there was no statistically significant effect of message framing on any of the three dependent variables. Message framing consistently explained less than 1% of the variation in the outcome variables and the critical *F* statistics were all well-below 3.85, which were the minimum reliably detectable thresholds indicated by our sensitivity analyses. Thus, if a statistically significant effect could be detected with a larger sample, it would still likely be negligible.

**TABLE 3:** Results from a series of 2 × 2 factorial ANCOVAs, using message framing and information safety to predict post-experiment perceptions of a COVID-19 contact tracing application, controlling for pre-experiment likelihood to download, in Study 1.

	Sum of squares	*df*	Mean square	*F*	*p*	η_*p*_^2^
**Intention to download**						
Pre-experiment likelihood	691.41	1	691.41	1833.54	<0.001	0.62
Message framing	0.04	1	0.04	0.10	0.75	0.00
Information safety	4.29	1	4.29	11.38	0.001	0.01
Message framing * Info Safety	0.06	1	0.06	0.15	0.70	0.00
Residuals	418.19	1109	0.38			
**Government should fund**						
Pre-experiment likelihood	410.83	1	410.83	657.15	<0.001	0.38
Message framing	0.57	1	0.57	0.91	0.34	0.00
Information safety	1.15	1	1.15	1.85	0.18	0.00
Message framing * Info Safety	0.00	1	0.00	0.01	0.93	0.00
Residuals	681.44	1090	0.63			
**Recommend to others**						
Pre-experiment likelihood	516.31	1	516.31	975.13	<0.001	0.47
Message framing	0.47	1	0.47	0.88	0.35	0.00
Information safety	5.72	1	5.72	10.80	0.001	0.01
Message framing * Info Safety	0.00	1	0.00	0.004	0.95	0.00
Residuals	582.42	1100	0.53			

##### High information safety versus low information safety

Table 3 shows a statistically significant effect of information safety on two of the three outcomes: intention to download the application and intention to recommend the COVID-19 contact tracing application to friends, family, and colleagues. According to the means in Table 2, participants in the high information safety conditions reported higher intentions to download and to recommend it than those in the low information safety conditions. There was no effect of information safety on perceptions of the application as a worthwhile use of government resources.

##### Interaction between message framing and information safety

As shown in Table 3, there were no statistically significant interactions between message framing and information safety in the prediction of any outcomes. The effect of information safety was evident regardless of autonomy-supportive or controlling message framing.

### Discussion

The aim of Study 1 was to assess two elements of social messages, and their effects on people’s intentions to abide government requests to use contact tracing technology. We found support for our hypotheses regarding information safety, indicating that data and information safety assurances may be vital tools in promoting the uptake of COVID-19 contact tracing applications. However, we did not find an effect of message framing or an interaction between information safety and message framing in the prediction of contact tracing application uptake. Belonging to the two message framing conditions (autonomy-support and control) did not reliably predict any of the three dependent variables. Meanwhile, belonging to the high information safety conditions resulted in a greater likelihood of downloading the application and of recommending it to friends and family, compared to the low information safety conditions. However, information safety did not affect people’s perceptions of the COVID-19 tracing application as a worthwhile use of government resources. The effects of information safety assurances were evident regardless of message framing condition.

In Australia, where the sample was collected, our survey was administered proximal to the actual launch of Australia’s contact tracing application, COVIDsafe. We collected data over a 72-h period basing the application description on contemporaneous media reporting and government press conferences. Three days after the data were collected, the government actually released the application and encouraged Australians to download it. Thus, participants likely had prior exposure to the government’s aims and rationale. Nonetheless, our conditions making information safety explicit enhanced participants’ willingness to use the application.

Study 1 leaves some possibilities unaddressed, and thus requires expansion. Our use of an Australian sample is a potential limitation because social and media discussion regarding contact tracing applications had become commonplace prior to our study. Thus, participants’ views of the technology had likely already, at least partially, developed. Accordingly, replication and expansion of this study in a country yet to implement contact tracing application technology may be more appropriate for testing our hypotheses, such was our aim in Study 2.

## Study 2

In Study 2, we sought to replicate our message framing and information safety results from Australia in a sample from another country that was yet to fully launch a contact tracing application. We selected the United States, which at the time of the data collection was experiencing an increase in COVID-19 cases, and had no uniform contact tracing policy. In addition, compliance with COVID-related prevention measures in the U.S. was highly variable, and popularly reported to be associated with differences in political affiliation, as well as trust in government health messaging. Thus, in addition to replicating our results in a different national climate, we also assessed additional variables to tap the unique U.S. climate in relation to compliance with COVID-19 prevention behaviors.

First, in the U.S. sample, we assessed perceived legitimacy of government, expecting that perceived legitimacy would be positively associated with more willingness to accept contact tracing across conditions, measured in both pre-and post-experimental manipulation assessments. We expected that, in the diverse political landscape in the United States, there would be varied perceptions of government messaging as legitimate, allowing us to examine a possible positive relationship between perceptions of the government as legitimate and intention to download and use a contact tracing application. Moreover, by measuring and including perceptions of government legitimacy, we were able to test for the independent effects of our experiment on intended application uptake, controlling for positive government perceptions.

Second, although not a theoretically derived question, given the potential relevance of political party affiliation to perceptions of government legitimacy, we also collected participants’ political affiliations, and examined differences across self-reported political groups in terms of their intended uptake. Again, given that we have no theory-based predictions regarding intended uptake and political affiliation, we examined these variables in an exploratory way, and for descriptive purposes only.

### Method

#### Participants

The sample comprised 888 U.S. adults, recruited by the professional survey company Qualtrics. Participants completed the survey online. We again conducted sensitivity analyses (Perugini et al., 2018) in G^∗^Power (Faul et al., 2007), to assess the minimum detectable effect size given our sample size, an alpha level of 0.05, power of 0.80, and two covariates (prior likelihood to download the application and perceived government legitimacy). The results suggested that our *N* of 888 was sufficient to reliably detect effects as small as η_*p*_^2^ = 0.01 (reflected in a critical *F* statistic of 3.85, the same as in Study 1). G^∗^Power derives an *f* statistic effect size in sensitivity analysis (in this case the *f* value was 0.094), so we used the formula reported in Cohen (1988, p. 281) to convert *f* to η_*p*_^2^ (simply *f*^2^), and rounded to the second decimal place.

The sample ranged in age from 18 to 90 years (*M* = 46.09, *SD* = 17.00), and included 359 males, 525 females, and four individuals who reported their gender as “other.” Participants’ political affiliations were relatively balanced across the sample, with 274 reporting as Republicans, 347 as Democrats, 235 as independents, 10 as libertarians, eight as greens, and 14 as “other.” The participant numbers in the libertarian, green, and “other” categories were too small to be statistically useful, therefore, these responses were changed to NA such that political affiliation could be used as a three-level factor variable comprising Democrats, independents, and Republicans.

#### Materials

In Study 2, we presented the same experimental survey materials as we did in Study 1. At the pre-experiment time point, we assessed participants’ likelihood of using a contact tracing application, and post-experiment we measured participants’ perceptions of the COVID-19 contact tracing application using the same three questions as in Study 1.

##### Post-experiment items

###### Perceived government legitimacy

In Study 2, we also included post-experiment questions that assessed participants’ perceptions of governmental authority as being legitimate using three items, “In general, I trust the government to do the right thing,” “I believe the government adequately represents the people,” and “I think messages from the government are trustworthy and reliable,” each answered on a 0 (not at all true of me) to 6 (Completely true of me) scale. Cronbach’s alpha of 0.95 indicated high internal consistency among these items, so we averaged the three items and used a single composite score.

#### Experimental Manipulation

As in Study 1, after answering the pre-experiment questions, participants were randomly assigned to one of four conditions: autonomy-supportive message framing with high information safety (*n* = 225), autonomy-support with low information safety (*n* = 229), controlling message framing with high information safety (*n* = 224), and control with low information safety (*n* = 210). The experimental manipulation was employed using the same materials as reported above in Study 1, gently edited for the American context (i.e., we removed “Australia” and replaced with “America”).

### Results

#### Preliminary Analyses

Means, standard deviations, and inter-correlations between the study variables are included below in Table 4. As we found in Study 1, the correlations between pre-experiment intentions to download a COVID-19 contact tracing application and post-experiment intentions, and positive post-experiment perceptions of a contact tracing application were positive. As expected, perceived government legitimacy correlated strongly and positively with positive perceptions of the COVID-19 contact tracing application.

**TABLE 4:** Inter-correlations, means, and standard deviations for the variables in Study 2.

	1	2	3	4	5
1. Pre-test likelihood	–				
2. Post-test intentions	0.82***	–			
3. Post-test government should fund	0.68***	0.81***	–		
4. Post-test recommend to others	0.75***	0.88***	0.87***	–	
5. Perceived government legitimacy	0.41***	0.41***	0.38***	0.42***	–
Mean	3.66	4.14	6.01	5.30	3.67
SD	3.69	3.70	3.54	3.71	1.88

Note. ***p < 0.001. Pre-test, pre-experiment; Post-test, post-experiment.

There were no differences across the four groups in (1) pre-experiment likelihood of downloading the COVID-19 contact tracing application, *F*(3,884) = 0.40, *p* = 0.76, η_*p*_^2^ = 0.001, (2) mean age, *F*(3,884) = 0.34, *p* = 0.80, η_*p*_^2^ = 0.001, or in (3) perceptions of the government as legitimate, *F*(3,884) = 0.45, *p* = 0.71, η_*p*_^2^ = 0.001. We used forced choice responding throughout the online survey, so there were no missing responses in the dataset. All continuous variables were standardized prior to analysis.

#### Primary Analyses

##### Experimental effects

To test if the total participant reports of likelihood of downloading the COVID-19 contact tracing application increased from pre- to post-experiment, we conducted a paired samples *t*-test. The results indicated that intentions to download the application increased from pre- (*M* = 3.66, *SD* = 3.69) to post-experiment (*M* = 4.14, *SD* = 3.70), *t*(887) = -6.57, *p* < 0.001 [95% CI -0.63, -0.34], Cohen’s *d* = 0.22. We also examined pre- to post-experiment intentions to download the application in each experimental group. Three of the four groups reported a small to moderately-sized increase in intention to download from pre- to post-experiment: (1) the autonomy-support plus high information safety group, *t*(224) = -2.41, *p* = 0.02 [95% CI -0.59, -0.06], Cohen’s *d* = 0.16, (2) the control plus high information safety group, *t*(223) = -5.73, *p* < 0.001 [95% CI -1.32, -0.64], Cohen’s *d* = 0.38, and (3) the control plus low information safety group, *t*(209) = -3.27, *p* = 0.001 [95% CI -0.72, -0.18], Cohen’s *d* = 0.23. There was no reliably detectable difference between pre- and post-experiment intentions to download in the autonomy-support plus low information safety group, *t*(228) = -1.40, *p* = 0.16 [95% CI -0.47, 0.08], Cohen’s *d* = 0.23 (see Table 5 for group-specific means and standard deviations).

**TABLE 5:** Experimental group-specific means and standard deviations for the pre-experiment (pre-test) and post-experiment (post-test) measures.

	Aut + Safe	Aut + Not Safe	Cont + Safe	Cont + Not Safe
Pre-test likelihood	3.74 [3.66]	3.83 [3.68]	3.52 [3.69]	3.52 [3.73]
Post-test intentions	4.15 [3.66]	3.94 [3.56]	4.50 [3.81]	3.97 [3.76]
Post-test support	6.19 [3.62]	5.88 [3.54]	6.26 [3.50]	5.68 [3.50]
Post-test recommend	5.59 [3.86]	5.03 [3.58]	5.62 [3.76]	4.92 [3.60]
Pre-post difference	0.32 [2.02]	0.20 [2.13]	0.98 [2.56]	0.45 [1.98]

*Note. Aut, autonomy-supportive message framing condition; Cont, controlling message framing condition; Safe, high information safety condition; Not Safe = low information safety condition; Pre-test likelihood [to download the application]; Post-test intentions [to download the application]; Post-test support [the government investing in the application]; Post-test recommend [the application to friends, family, and colleagues]; Pre-post difference, post-test intentions minus pre-test likelihood.*

Next, to examine the role of message framing and information safety in predicting group differences on the post-experiment measures, we ran the same 2 × 2 factorial ANCOVAs as we did in Study 1, using the two (message framing and information safety) two-level (autonomy versus control and high information safety versus low information safety) factorial predictors. First, we predicted post-experiment intention to download the application. Second, we predicted post-experiment perceptions of the application as a worthwhile use of government resources. Third, we predicted post-experiment intention to recommend the application to friends and family. In all three models, we controlled for self-rated initial likelihood of downloading the application. In addition, given the substantial positive correlations between perceived government legitimacy and intention to download the application, we included perceived government legitimacy as a covariate in the models.

###### Autonomy-supportive versus controlling message framing

**FIGURE 2:**
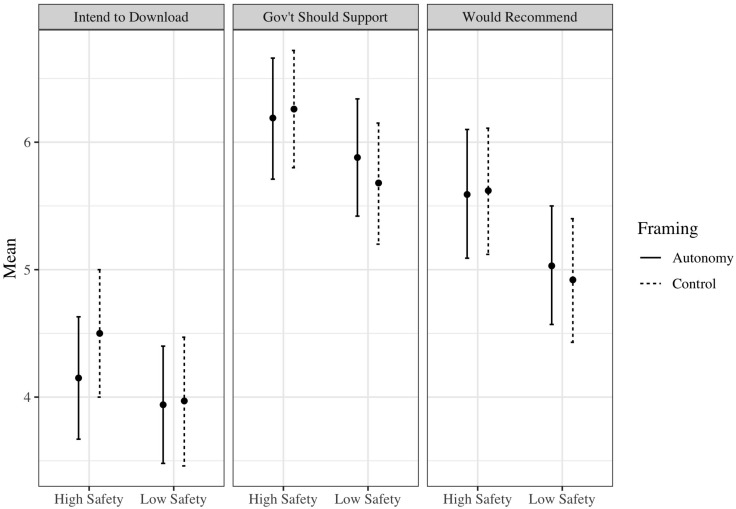
Mean scores and 95% confidence interval for the three dependent variables according to message framing condition (autonomy or control) by information safety condition (high or low) in Study 2.

The experimental group-specific means presented in Table 5 (and illustrated in Figure 2), coupled with the 2 × 2 factorial ANCOVA results shown in Table 6, demonstrate that there was a statistically significant main effect of message framing for one of the three dependent variables. Counter to expectations, in the prediction of post-experiment intentions to download the COVID-19 contact tracing application, participants in the controlling message framing conditions reported higher intention to download the application than those in the autonomy-supportive message framing conditions. There was no reliably detectable main effect of message framing on perceptions of the application as a worthwhile use of government resources or post-experiment intention to recommend the application to friends and family.

**TABLE 6:** Results from a 2 × 2 factorial ANCOVA, using message framing and information safety to predict post-experiment perceptions of a COVID-19 contact tracing application, controlling for pre-experiment likelihood to download.

	Sum of squares	*df*	Mean square	*F*	*p*	η_*p*_^2^
**Intention to download**						
Pre-experiment likelihood	598.77	1	598.77	1908.68	<0.001	0.68
Perceived gov’t legitimacy	6.48	1	6.48	20.64	<0.001	0.02
Message framing	2.66	1	2.66	8.47	0.004	0.01
Information safety	1.65	1	1.65	5.26	0.02	0.01
Message framing * Info Safety	0.75	1	0.75	2.39	0.12	0.00
Residuals	276.69	882	0.31			
**Government should fund**						
Pre-experiment likelihood	412.61	1	412.61	791.30	<0.001	0.47
Perceived gov’t legitimacy	10.85	1	10.85	20.81	<0.001	0.02
Message framing	0.19	1	0.19	0.36	0.55	0.00
Information safety	2.80	1	2.80	5.37	0.02	0.01
Message framing * Info Safety	0.64	1	0.64	1.23	0.27	0.00
Residuals	459.91	882	0.52			
**Recommend to others**						
Pre-experiment likelihood	497.92	1	497.92	1189.78	<0.001	0.57
Perceived gov’t legitimacy	14.03	1	14.03	33.53	<0.001	0.04
Message framing	0.35	1	0.35	0.84	0.36	0.00
Information safety	5.29	1	5.29	12.64	<0.001	0.01
Message framing * Info Safety	0.29	1	0.29	0.68	0.41	0.00
Residuals	369.12	882	0.42			

###### High information safety versus low information safety

As Table 6 shows, there was a statistically significant effect of information safety on all three outcome variables. Coupling the results from Table 6 with the means in Table 5, belonging to the high information safety conditions resulted in more positive perceptions of the application belonging to the low information safety conditions.

###### Interaction between message framing and information safety

As in Study 1, and as shown in Table 6, there were no statistically significant interactions between message framing and information safety in the prediction of any outcomes.

###### Perceived government legitimacy

Perceptions of government power as legitimate were an independent and statistically significant positive predictor of post-experiment intentions to download the COVID-19 contact tracing application, perceptions of the application as a worthwhile use of government resources, and post-experiment intention to recommend the application to friends and family.

#### Political Affiliation

Using ANOVAs, we compared participants who self-reported as Democrats, Republicans, and independents on their: (1) pre-experiment intention to download a COVID-19 contact tracing application and (2) perception of the government as legitimate. When using post-experiment intentions to download the COVID-19 tracing application as the outcome, there was a small statistically significant main effect of political affiliation, *F*(2,853) = 4.39, *p* = 0.01, η_*p*_^2^ = 0.01. *Post hoc* comparisons using Tukey’s HSD indicated that independents (*M* = 3.18, *SD* = 3.36) reported lower intentions to download the application when compared to democrats (*M* = 4.10, *SD* = 3.70). There were no differences between democrats and republicans (*M* = 3.69, *SD* = 3.93), or between republicans and independents. In the prediction of perceived government legitimacy, there was also a statistically significant main effect of political affiliation, *F*(2,853) = 48.98, *p* < 0.001, η_*p*_^2^ = 0.10. Tukey’s HSD *post hoc* comparisons showed Republicans (*M* = 4.57, *SD* = 1.87) reported higher perceived legitimacy than both Democrats (*M* = 3.35, *SD* = 1.77) and independents (*M* = 3.19, *SD* = 1.69), who did not differ.

## General Discussion

The COVID-19 pandemic presents scientists and public health experts with the challenge of motivating wide-spread and substantial behavior change quickly and reliably. As a specific example of behavior change, we explored several variables expected to relate to, or impact, people’s intention to use contact tracing technology to contain outbreaks of the virus. As is the case with many variables in psychology, these studies demonstrate that the best predictor of people’s future intentions to download and use a COVID-19 contact tracing application is their prior intentions. However, across large and representative samples from two countries, we also show that specific public messaging strategies can increase intended behavior change, even when accounting for prior intentions. The results suggest that the provision of data and information safety assurances may be key in affecting people’s future use of contact tracing technology, an effect we found in both samples regardless of whether messaging was framed in an autonomy-supportive or controlling manner, as well as independent of prior intentions and perceptions of the government as legitimate.

### Information Safety and Government Perceptions

Across Studies 1 and 2, we found that information safety assurances had meaningful effects on favorable perceptions of contact tracing applications. In Study 2, we tested the degree to which positive perceptions of contact tracing applications were a function of perceived government legitimacy. We found that—while perceptions of the government as legitimate were strongly associated with intended application uptake—high levels of information safely continued to be a meaningful predictor of intended application uptake, even controlling for government perceptions. These findings thus highlight the importance of transparency in source codes, and explicit protections regarding data accessibility, to ameliorate people’s concerns with controlling surveillance when implementing such potentially life-saving technologies.

Of course, we would be remiss if we did not emphasize that messages about data security should be anchored in truth. If the public is assured that personal data are safe, the information needs to actually be protected. We would expect that if information safety messages originated from an untrustworthy government or entity, the ability of the message to instigate behavior change would likely be nullified. Indeed, the strong and positive effects of perceived legitimacy suggest that governments should seek to maintain integrity with regards to their public messaging, in order to maintain the public trust required to sustain COVID-19-related behavior change and compliance over the long term.

### Message Framing

Across six models (three per study), we assessed the effects of autonomy-supportive and controlling message framing on intended application uptake, perceptions of the application as a worthwhile government investment, and likelihood of recommending to friends. In five of these six models, there was no reliably detectable effect of message framing. The lone main effect was in the prediction of intended uptake in Study 2. Counter to our expectations, the result suggested that belonging to the controlling message framing conditions resulted in increased likelihood to download the contact tracing application after the experiment compared to the autonomy-supportive conditions. While we are reluctant to attach too much weight to a single statistically significant effect in a batch of six, the result may suggest that people could be responsive to firmer messaging in the face of confusion and mortal threat, such as people in the United States, as well as other parts of the global population, currently face. Indeed, while autonomy support is often demonstrated to effectively initiate behavior change, in the context of a global pandemic, messaging that provides rationales and choice points needs to be balanced with the fact that behavior change is essential, not optional, to maintain public health.

Important to note is that behavior can be initiated for both autonomous *and* controlled reasons and, in the main, our results showed that, when paired with safety reassurances, participants exposed to *either* autonomy-supportive or controlling message framings increased in their intention to engage with contact tracing technology. Sources of external pressure or feelings of internal pressure like guilt and shame can effectively motivate short-term behaviors (Pelletier et al., 2001). Where such controlled forms of motivation tend to lack efficacy is in their ability to sustain behavior change over the long term (Ryan et al., 2008; Ng et al., 2012). Given that downloading a contact tracing application is a single instance behavior, firmer language, coupled with information safety assurances, may have utility. It would, however, be useful to examine the effects of autonomy-supportive and controlling message framing on the maintenance of behavior change longitudinally, especially with hard to sustain behaviors such as social distancing or frequent hand washing.

### Political Affiliation

For exploratory purposes, we examined differences between participants at the level of self-reported political party affiliations. Independents reported the lowest intentions to download the contact tracing application, and significantly differed from Democrats, who reported the most. The differences in level of endorsement across political affiliates suggest that a one-size-fits all messaging strategy may not be useful, given people’s existing political ideologies appear related to their intention to abide government recommendations. Messaging tailored to meet specific political party values may be useful, though the claim is speculative until future research tests such a proposition.

### Limitations, Future Directions, and Conclusion

Our use of an Australian sample in Study 1 is a potential limitation because social and media discussion regarding contact tracing applications had been widespread for several weeks prior to our study. Thus, participants’ perceptions of contact tracing had likely already formed. More light would have been shed on this possibility had we included a neutral control group, which we did not, and should be included in future studies. In addition, in both studies, participants in all experimental conditions were provided with a description of the application, including how it can accelerate contact tracing. Given all groups increased their willingness to use the application, our description may have provided all participants with a self-evident, value-aligned rationale, which according to SDT, would facilitate internalization and intent. Indeed, rationale provision is a key element of autonomy-supportive leadership (Ryan and Deci, 2017). Ancillary correlations reported in Online Supplementary Materials S2, S3 demonstrated that people’s willingness to engage with contact tracing technology was strongly associated with the belief that a contact tracing application has value, is safe, and would benefit both self and others. Future studies of public messaging strategies and behavior change would be well-served if conducted in countries without prior social discussion regarding contact tracing applications, and if the rationale component was not presented to participants in controlling message framing conditions.

Also, our results may only generalize to computer literate individuals because the online nature of the survey required access to and knowledge of computer and mobile phone technologies. This highlights a limitation not of our study, but of contact tracing technology in general: access to contact tracing technology may not be equitable across all groups. People who are not technologically literate and those who do not use smartphones and applications may not directly benefit from use of a contact tracing application. The obstacles to application use may apply particularly to groups that are vulnerable to COVID-19 such as the elderly, but could extend to other groups such as children and groups without the cognitive or physical capacities required to use the application. If community uptake of contact tracing is widespread, individuals without access to contact tracing applications will likely benefit from their use indirectly because those with COVID-19 will know to self-isolate more quickly. However, governments and policy makers should consider how vulnerable groups can better access the features of contact tracing applications, without smartphone use or knowledge.

Taken together, our studies identified meaningful discursive strategies relevant to COVID-19-related public health messaging. In particular, assurances regarding information safety and non-surveillance were key. However, our results also suggest that message framing and information safety assurances alone, are not sufficient to cultivate the necessary change to existing attitudes toward contact tracing applications. People’s current perceptions of contact tracing relate to their future intentions to use such technology. Therefore, messaging designed to debunk existing contact tracing-related qualms may be useful, especially if combined with true freedom from surveillance and privacy built in to technology design (Calvo et al., 2020). In addition, people’s perceptions of government legitimacy and political affiliations relate to their intended uptake of contact tracing technology. Therefore, governments should strive for clarity and consistency to maintain public trust, and messaging could be more useful if tailored to suit specific political party values. Questions regarding the ability of social messages to affect behavior are more crucial now than it has ever been, and we hope to spur more research examining these effects.

## Data Availability Statement

The datasets presented in this study can be found in online repositories. The names of the repository/repositories and accession number(s) can be found below: https://osf.io/u5x3r/.

## Ethics Statement

The studies involving human participants were reviewed and approved by the Monash University Human Research Ethics Committee (ref # 23854) and the Australian Catholic University Human Research Ethics Committee (ref # 2020-65R). The patients/participants provided their written informed consent to participate in this study.

## Author Contributions

EB, RR, MN, and RC developed the study design. EB, RR, MN, AS, PS, and EG developed the stimulus materials and assessments. EB and RR preregistered the study. AS and PS collected the data for Study 1. EB and RR collected the data for Study 2. MN cleaned the data for Study 1. EB cleaned the data for Study 2, conducted the analyses for both studies, and led preparation of the manuscript. All authors provided critical feedback on the analyses, contributed to editing and revising the manuscript, and approved it for submission.

## Conflict of Interest

The authors declare that the research was conducted in the absence of any commercial or financial relationships that could be construed as a potential conflict of interest.
