# Supplementary material for: Information Safety Assurances Increase Intentions to Use COVID-19 Contact Tracing Applications, Regardless of Autonomy-Supportive or Controlling Message Framing
Source: Front Psychol. 2021 Jan 7;11:591638. doi: 10.3389/fpsyg.2020.591638 (PMC7852485; doi:10.3389/fpsyg.2020.591638)
Supplement: Supplementary file 1 [file Table_1.DOCX]

**Online Supplementary Materials**

This document contains supplementary materials for the manuscript:

**Information safety assurances increase intentions to use COVID-19 contact tracing applications, regardless of autonomy-supportive or controlling message framing**

Emma L. Bradshaw^1^, Richard M. Ryan^1^, Michael Noetel^2^, Alexander K. Saeri^3^, Peter Slattery^3^, Emily Grundy^4^, & Rafael Calvo^5^

^1^Institute for Positive Psychology and Education, Australian Catholic University

^2^School of Health and Behavioural Sciences, Australian Catholic University

^3^Monash Sustainable Development Institute, Monash University

^4^School of Psychology, Deakin University

^5^Dyson School of Design Engineering, Imperial College London

S1.

*Experimental manipulation vignettes for the autonomy-support, control, information is safe, and information is not safe experimental conditions*

**Introduction for All Conditions**

In this section, you will read about one possible version of a COVID-19 trace app based on Australian media reporting and Australian government press conferences. Similar apps or the use of mobile phones to trace COVID-19 have been proposed or deployed in other countries. At the end of the survey, you will receive links to media and government information about the planned Australian app.

In the midst of this global pandemic, one of the most effective ways to stop the spread of COVID-19 is to trace the contacts of people who are infected, and test those contacts. This typically involves interviewing the infected person to identify possible contacts. The government has commissioned the development of a digital contact tracing app to speed up this process. It works like this

1. People download the COVID-19 trace app on their mobile phone.
2. The COVID-19 trace app keeps a record of mobile phones you have been in close contact with (within 1.5m for at least 15 minutes).
3. When a person tests positive for COVID-19, health professionals use the data from their app and to notify people who have been in close contact with them, so they can get tested or self-isolate.

**Autonomy-Support Condition**

Downloading the COVID-19 trace app means you are allowing information about who you have come into close contact with to be electronically monitored, which may feel intrusive. The reason for this unusual measure is that it is the most effective way to help people find out about their risk if they have come in contact with an infected person. Doing so means they can then make the right choices to protect themselves and their loved ones. That is why it’s hoped that you will choose to participate in this important program. Using the app is entirely voluntary. You have the choice to download and to activate, and you can opt out at any time. Making this choice is a way you can really contribute to containing the spread. (128 words)

**Controlling Condition**

Downloading the COVID-19 trace app means you are allowing information about who you have come into close contact with to be electronically monitored. Even if it feels intrusive, this is something people shouldn’t question, because it is clearly the most effective way for authorities to track who has been in contact with an infected person. You need to help authorities notify those at risk of contracting the virus. Given the current threat, we think you must do this to be a responsible citizen. Downloading the app is not really a choice—it’s a thing that you should just do. To comply with this program, you should download the app and ensure that it’s activated. Complying with this requirement is the best way to stop the spread of the virus. (128 words)

**Information is Safe Condition**

Information from the COVID-19 trace app will be stored locally on a phone, encrypted, and only transferred to a health data bank if a person tests positive for COVID-19. Once there, data cannot be accessed by any other parties, private or governmental, and will not be used for any other purposes. The app is designed so that your personal identity and personal information are protected. Data will be destroyed every 21 days so that it cannot be used later by anyone, for any reason. (84 words)

**Information is Not Safe Condition**

Information from the COVID-19 trace app will be stored locally on a phone and then transferred to a health data bank for use in tracing the contacts of a person who tests positive for COVID-19. Once there, the data will be owned by the government and may be accessed for other important purposes. The app is designed so that the data can be stored long-term and it is possible that the data will be used in later analyses for other health or government purposes. (84 words)

S2

*Post-test measurements related to basic psychological need satisfaction and goal contents*

When it comes to the COVID-19 tracing app, how true are the following of you? (0-10)

- Autonomy
  - I see the value in using the app
- Safety
  - I trust that the information in the app will be kept secure
- Competence (reverse scored)
  - Using the app sounds difficult
- Beneficial to others
  - Using the app would help protect my community
- Beneficial to oneself
  - Using the app would help me protect myself from other people

S3.

*Inter-correlations between the main study variables (1-4) and five additional post-test items presented to participants*

|  | 1 | 2 | 3 | 4 | 5 | 6 | 7 | 8 | 9 |
| --- | --- | --- | --- | --- | --- | --- | --- | --- | --- |
| 1. Pre-test likely | - |  |  |  |  |  |  |  |  |
| 2. Intentions to download | 0.79*** | - |  |  |  |  |  |  |  |
| 3. Support | 0.61*** | 0.77*** | - |  |  |  |  |  |  |
| 4. Intentions to recommend | 0.68*** | 0.85*** | 0.81*** | - |  |  |  |  |  |
| 5. Autonomy | 0.60*** | 0.73*** | 0.80*** | 0.76*** | - |  |  |  |  |
| 6. Safety | 0.58*** | 0.69*** | 0.74*** | 0.76*** | 0.71*** | - |  |  |  |
| 7. Competence | -0.04 | -0.05 | -0.04 | 0.02 | -0.10*** | 0.08** | - |  |  |
| 8. Beneficial to self | 0.57*** | 0.71*** | 0.80*** | 0.74*** | 0.87*** | 0.71*** | -0.08** | - |  |
| 9. Beneficial to others | 0.59*** | 0.71*** | 0.73*** | 0.71*** | 0.79*** | 0.70*** | -0.01 | 0.82 | - |
| M | 3.96 | 4.6 | 5.85 | 4.94 | 6.01 | 4.79 | 3.7 | 6.19 | 5.63 |
| SD | 3.49 | 3.57 | 3.05 | 3.38 | 2.98 | 3.38 | 2.93 | 2.88 | 3.08 |
